# Molecular Characteristics, Heterogeneity, Plasticity, and Cell of Origin of Neuroendocrine Bladder Cancer

**DOI:** 10.47248/chp2502010005

**Published:** 2025-03-06

**Authors:** Dongbo Xu, Qiang Li

**Affiliations:** 1. Department of Urology, Roswell Park Comprehensive Cancer Center, Buffalo, NY 14203, USA;; 2. Department of Pharmacology & Therapeutics, Roswell Park Comprehensive Cancer Center, Buffalo, NY 14203, USA

**Keywords:** neuroendocrine bladder cancer, small cell bladder cancer, molecular characteristics, heterogeneity, plasticity, cell of origin

## Abstract

Neuroendocrine bladder cancer (NEBC) is a rare but highly aggressive cancer, representing approximately 1% of urinary bladder cancer. The most common NEBC is small cell bladder cancer (SCBC), characterized by high rates of recurrence, chemotherapy resistance, and early mortality. SCBC is histologically identical to small cell lung cancer (SCLC) but remains significantly understudied. Advances in next-generation sequencing techniques have partially elucidated the molecular characteristics of NEBC and identified druggable targets. This review compiles recent studies on human NEBC samples, summarizing key findings on their genomic alterations and molecular subtyping. Notably, it highlights specific mutations in the *TERT* promoter and epigenetic modifiers in NEBC, as well as molecular subtyping based on lineage-specific transcription factors, including ASCL1, NEUROD1, and POU2F3. Furthermore, this review explores the significant tumor heterogeneity and cellular plasticity observed in NEBC and discusses its cell of origin and potential therapeutic targets (MET inhibitor or DLL3) identified by preclinical NEBC models. Emerging evidence suggests that NEBC may share a common origin with urothelial carcinoma (UC), arising from a UC precursor. Advancing our understanding of NEBC tumorigenesis and identifying druggable targets will enhance treatment outcomes for patients with NEBC.

## 1. Introduction

Bladder cancer (BC) is the fourth most common cancer in males in the United States, with an estimated 83190 new cases and an estimated 16840 deaths in 2024 [1]. Almost 90% of bladder cancers are urothelial carcinoma (UC). Approximately 25–30% of muscle-invasive bladder cancers (MIBC) exhibit divergent differentiation, such as squamous, glandular, adenocarcinoma, micropapillary, plasmacytoid, trophoblastic, and neuroendocrine (NE), *etc*. [2]. According to the WHO classification of Urinary and Male Genital tumors, neuroendocrine bladder cancer (NEBC) represents ~1% of urinary BC, including small cell neuroendocrine carcinoma (also known as small cell bladder cancer, SCBC), large cell neuroendocrine carcinoma (LCNEC), well-differentiated neuroendocrine tumor, and paraganglioma [3,4]. SCBC represents the most common NEBC [3,5]. The first reported case of SCBC was described by Cramer *et al*. in 1981 [6]. SCBC is a rare and highly aggressive cancer with a poor prognosis and a lack of standardized treatment options. The therapeutic management of SCBC is empirically guided by clinical strategies and insights derived from small cell lung cancer (SCLC) [7–9]. The rarity of SCBC limits genomic, biological, and clinicopathological research, leaving patients with limited treatment options beyond the standard chemotherapy regimen of Etoposide and Cisplatin. Despite significant advances in the basic understanding over the past decades, SCBC remains understudied, with challenges in uncovering its pathogenesis, tumor heterogeneity, plasticity, cell of origin, and potential therapeutic targets.

Numerous studies have reviewed the epidemiology, clinical presentation, diagnosis, treatment, and prognosis of SCBC. Identified risk factors for SCBC include smoking, male sex, and advanced age [10]. SCBC is observed five times more frequently in men than in women, with cigarette smoking history in 50–70% of patients [11–13]. The average age of onset for SCBC is 72 years [14]. The most common symptom is painless gross hematuria (67–100% in SCBC) with or without dysuria [15]. Other symptoms include urinary obstruction, abdominal pain, pelvic pain, recurrent urinary tract infection, or weight loss [11,16–18]. The diagnosis of SCBC is based on histopathological examination of tumor tissue obtained by cystoscopy and transurethral resection of the bladder tumor (TURBT) or radical cystectomy [19]. The current clinical management of SCBC includes chemotherapy as the first approach, along with radical resection, radiotherapy, or multimodality treatment strategies [10,20]. Second-line immunotherapy demonstrates limited efficacy in the treatment of SCBC [21]. The overall prognosis for SCBC remains extremely poor. The median survival of SCBC patients ranges from 12 to 24 months with treatment, but only 4 to 5 months without treatment. The five-year survival rate varies between 8% and 40% [10,17,18,22]. Like SCBC, LCNEC is frequently presented with locally advanced and high-stage disease [23,24]. LCNEC has been treated using a similar approach to SCBC, involving neoadjuvant chemotherapy (NAC) followed by cystectomy [5]. In a cohort of 43 LCNEC patients and 192 SCBC patients, LCNEC patients demonstrated longer overall survival (OS) compared to those with SCBC only [25]. However, in another larger cohort of LCNEC (n = 80), LCNEC showed the worst disease-free survival compared to SCBC or mixed NEBC tumors in patients who underwent cystectomy [26].

**Figure 1:**
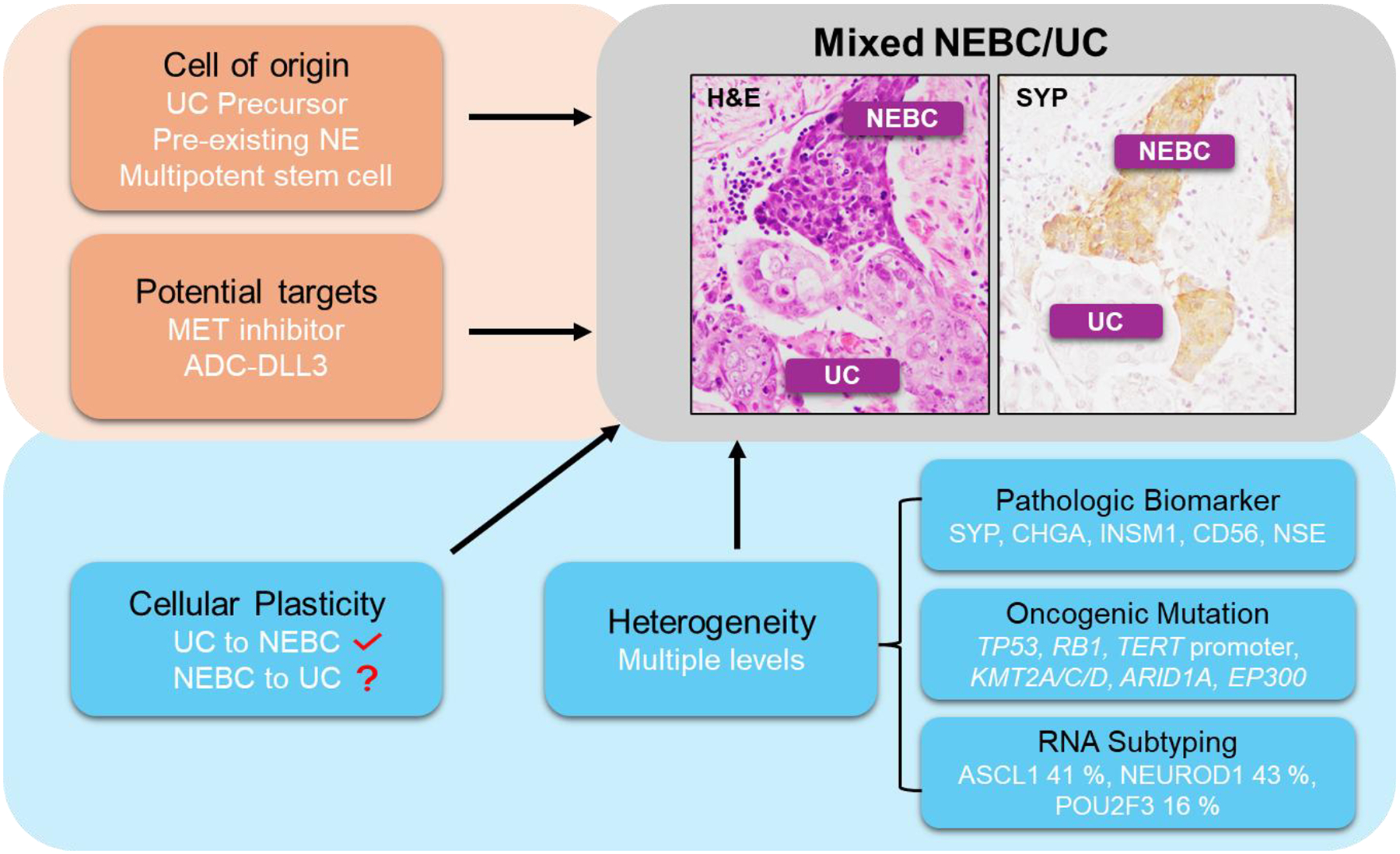
Schematic representation of the cell of origin, tumor heterogeneity, cellular plasticity, and potential therapeutic targets in NEBC. The diagram illustrates the diverse cancer cell populations observed in a mixed NEBC and UC tumor, as confirmed by hematoxylin and eosin (H&E) and immunohistochemical (IHC) staining of synaptophysin (SYP).

In this review, we summarize the molecular characteristics of NEBC and discuss its underlying tumor heterogeneity, cellular plasticity, cell of origin, and potential targets of NEBC or NE-like tumors (Figure 1). This review aims to enhance understanding of NEBC tumorigenesis and classification, guide the discovery of new therapeutic targets, and improve the effectiveness of treatment approaches.

## 2. Histopathology and Immunohistochemistry of NEBC

**Figure 2:**
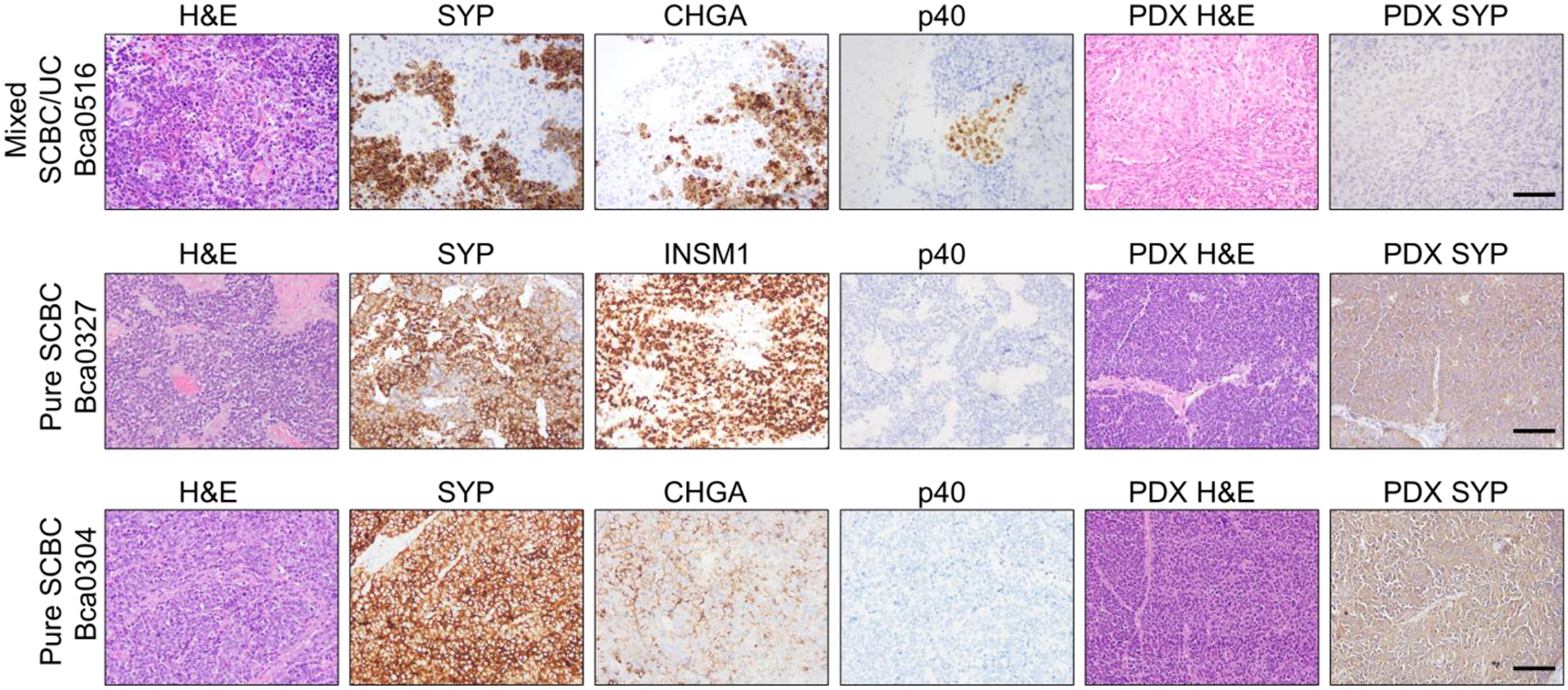
H&E and IHC staining of three SCBC tumors and corresponding PDX. Disassociated SCBC tumor cells were implanted into NSG mice. The Bca0516 primary tumor displayed mixed UC and SCBC components, characterized by a mixed histologic appearance and heterogeneous expression of UC and NE markers. Bca0516 PDX displayed only UC histology and was negative for NE marker (with SYP as the representative marker shown). In contrast, Bca0327 and Bca0304 were identified as pure SCBC tumors in both primary tumors and PDXs. NE markers: SYP, CHGA, INSM1; UC marker: p40. Scale bar: 100 μm.

The histological features of SCBC are almost identical to SCLC, sharing the same diagnostic criteria established by the WHO classification system [20]. Morphologically, SCBC exhibits small cells with a high nuclear-to-cytoplasmic ratio, nuclear molding, abundant mitotic figures, and necrosis. Immunohistochemical (IHC) staining with NE biomarkers, including chromogranin A (CHGA), SYP, CD56, neuron-specific enolase (NSE), and insulinoma-associated protein 1 (INSM1), assists in the diagnosis of NEBC [13,27,28]. Due to the 38–70% coexistence of SCBC with UC and its differentiated components, epithelial biomarkers such as cytokeratins, p63, p40, EMA, and CAM 5.2 are also utilized in its diagnosis [12,18,20,29]. Compared to SCBC, LCNEC comprises large, high-grade polygonal tumor cells that exhibit variable amounts of cytoplasm and prominent nucleoli [2]. LCNEC is also characterized by typical NE markers including CD56, synaptophysin, chromogranin, and INSM1 [30]. Figure 2 illustrates the histological features of three SCBC patients alongside their corresponding patient-derived xenografts (PDXs).

## 3. Genomic Alterations in NEBC

Next-generation sequencing has provided valuable insights into the mutational profiling of NEBC, offering genetic evidence to investigate its pathogenesis. SCBCs exhibit significant pathologic and genomic similarities to SCLC. Table 1 summarizes the common genetic alterations in NEBC. Like SCLC, SCBC frequently harbors alterations in *TP53* and *RB1*, with a high prevalence of co-alterations in both genes [7,31]. However, the *TERT* promoter was identified as a bladder-specific mutation with high frequency, which is absent from small cell carcinoma of other origins including lung [7,32,33]. The most frequent mutations in LCNEC include *TP53* (92%), *RB1* (65%), and *TERT* promoter (76%). Additionally, LCNEC harbors potentially targetable alterations in *ERBB2*, *ERBB3*, and *PIK3CA* at a frequency of 11% [26]. In another cohort of 18 LCNECs, mutations were identified in the *TERT* promoter (89%), *TP53* (100%), and *RB1* (100%), with 56% of tumors exhibiting co-alterations in *TP53* and *RB1* [25].

In a cohort of 61 SCBC patients from Memorial Sloan Kettering Cancer Center (MSKCC), the most frequently altered genes were *TP53* and *RB1*, found in 90% of patients, with co-alterations occurring in 80% [7]. A comparison study was conducted to identify similarities and differences in histology, cell lineage, and organ specificity between SCBC and SCLC tumors. The whole-exome and/or MSK-IMPACT sequencing identified a high prevalence of APOBEC-mediated mutational processes in SCBC, revealing a distinct pathogenic mechanism driving smoking-associated SCBC compared to SCLC. SCBC exhibited a significantly higher rate of biallelic mutations in *TP53* and *RB1* than UC. The coexistence of identical *TP53* and *RB1* mutations in UC suggests that these alterations alone are insufficient for the development of SCBC. SCBC-specific *TERT* promoter mutations were identified in 95% of patients but were absent in SCLC [33]. Moreover, mutations in epigenetic modifiers such as *KMD6A*, *ARID1A*, *CREBBP*, *EP300*, and *KMT2A/C/D* were identified in 74% of SCBC cases, resembling those found in UC but rarely observed in SCLC. These findings suggest that SCBC likely originates from a UC precursor. Evolutionary heterogeneity analysis of SCBC indicated that *TP53* and *RB1* are not the drivers of SCBC transformation from UC, but likely arose early in the molecular time, shortly after founding driver mutations. A subsequent genomic study from MSKCC analyzed 47 pre-treatment SCBC tumors in the neoadjuvant setting and correlated genomic alterations with treatment responses. *TP53* (87%), *TERT* promoter (83%), *RB1* (79%), *KMT2D* (28%), *ERCC2* (26%), *CREBBP* (23%), *ARID1A* (19%), *EP300* (15%) were altered frequently in SCBC [34]. Notably, *ERCC2* mutations were significantly enriched in patients who achieved complete responses following NAC, consistent with the findings observed in UC [35,36].

Another recent genomic study from MD Anderson Cancer Center analyzed 34 SCBCs and 84 conventional UCs along with a reference of 408 MIBCs in the TCGA (The Cancer Genome Atlas) database [37]. The mutational profiles revealed that *TP53* (93%) and *RB1* (47%) were mutated in SCBC at a higher frequency than in UC from the TCGA cohort. SCBC and UC tumors shared several significantly mutated genes, particularly in paired cases containing both SCBC and UC components. Identical mutations in *p53* (p.H179Y, p.C176F) and *RB1* (p.R798fs) observed in paired tumors suggested a clonal evolutionary process with a shared origin. Moreover, no SCBC tumors exhibited the typical mutations enriched in luminal conventional UC, suggesting that SCBC likely originates from a basal lineage.

In a larger study, 3.92% (132) of SCBC cases were identified among 3,368 UC samples. The three most frequent mutations were *TP53* (92%), *RB1* (75%), combined *TP53/RB1* (72%), and *TERT* promoter mutations (68%) [32]. Transcriptome RNA sequencing analysis of 24 pure SCBCs and 51 MIBC UCs revealed that SCBC expressed lower levels of canonical T-cell biomarkers, showed similar signature enrichment to SCLC, but displayed distinct gene expression profiles compared to UC. Similarly, a recent study of 44 SCBC cases (36 pure SCBCs and 8 mixed cases with small-cell components) identified frequent alterations, including *TP53* (75%), *RB1* (38%), and *TERT* mutations (92%), along with other mutations such as *ARID1A* (38%), *MYCL* (33%), and *PIK3CA* (21%) [38]. Another NEBC study included 103 SCBC and 19 LCNBC [39]. In 33 sequenced tumors, mutations in *TP53*, *RB1*, and *TERT* were detected in 30 (91%), 26 (79%), and 27 (82%) tumors, respectively. However, immunohistochemistry (IHC) results on the overall cohort indicated 93% (101/108) abnormal p53 staining and 92% (103/112) loss of Rb expression. The staining patterns of p53 and Rb did not show a significant difference in a paired analysis comparing NE and non-NE in the same tumor.

## 4. NE-like or Neuronal as a Subtype of MIBC

The ability to accurately differentiate BC subtypes can offer valuable prognostic insights and potentially predict therapeutic responses for precise patient stratification and personalized treatment approaches. Several subtype classification systems for MIBC have been reported over the past decade. The first molecular subtyping of MIBC was performed by a research group at Lund University [46]. Another research group at the University of North Carolina (UNC) used consensus clustering to divide MIBC into luminal and basal two subtypes [47]. Then, the Lund University group developed a newer classification system including five subtypes: urobasal A, genomically unstable, urobasal B, squamous cell carcinoma-like (SCCL), and an infiltrated class [48]. The same Lund group analyzed a cohort of 307 advanced bladder cancers by global gene expression, resulting in six consensus clusters [40]. Compared to the other known MIBC molecular subtypes clustering including UNC classification [47], MDA classification [49], and TCGA consortium [50], the six clusters were classified as urothelial-like, genomically unstable, epithelial-infiltrated, SCCL/mesenchymal-infiltrated, SCCL/UroB, and small-cell/neuroendocrine-like. Combined with IHC profiling, the cohort was divided into 5 tumor-cell phenotypes with molecular pathological definitions: urothelial-like, genomically unstable, basal/SCCL, mesenchymal-like, and small-cell/neuroendocrine-like [40]. This combination of molecular pathology (tumor-cell phenotype) and global mRNA profiling has been proposed for accurate subtyping of MIBC. This approach, known as the Lund taxonomy, was subsequently validated in a TCGA cohort [51].

A comprehensive analysis of the TCGA cohort of 412 MIBC cases identified five expression subtypes including luminal-papillary (35%), luminal-infiltrated (19%), luminal (6%), basal-squamous (35%), and neuronal (5%) [41]. Neuronal subtype includes 3 tumors with NE histology and 17 tumors without NEBC histologic features. All these tumors showed relatively high expression of neuronal differentiation genes and typical NE markers. However, the majority of these “neuronal” were not consistent with NEBC histologic features but were like conventional UC phenotypically, even though they have poor outcomes similar to NEBC.

Batista *et al*. analyzed transcriptome expression profiles of MIBC from seven institutions and developed a strict single-sample NE-like classifier by machine learning to identify NE-like tumors regardless of their pathologic presentation [43]. This classifier included an 84-gene panel and proved to be more stringent than previous subtyping models for NEBC. To validate the accuracy of the NE-like classifier, seven MIBCs with SCBC histology were 100% identified as NE-like. The study also utilized the CellMiner analysis tool to generate drug response scores, providing a numerical metric to predict chemotherapy responses for each NE-like tumor. NE-like tumors are predicted to be more sensitive to chemotherapy drugs, such as cisplatin, gemcitabine, and etoposide (agents commonly used in MIBC treatment), compared to basal and luminal tumors. A subsequent study validated this NE-like classifier and its ability to predict poor prognosis in a retrospective multicenter cohort of 234 patients treated with cisplatin-based NAC [52]. Ten patient samples were classified as the NE-like subtype with by strong expression of NE markers and the absence of basal or luminal markers. These patients exhibited significantly higher cancer-specific mortality rates compared to those with non-NE-like subtypes.

A comprehensive profiling study integrated six published MIBC classification systems from 1750 samples to develop a unified consensus subtyping framework. This system includes six subtypes: luminal papillary (24%), luminal non-specified (8%), luminal unstable (15%), stroma-rich (15%), basal/squamous (35%), and NE-like (3%) [44]. With the help of a single-patient classifier [43], this system enables the assignment of a consensus class to each patient, potentially facilitating more precise treatment strategies in the future. In this system, the NE-like class exhibited overexpression of neuroendocrine differentiation genes, lacked immune infiltration markers, was associated with the poorest prognosis, and displayed profiles suggestive of a potential response to radiotherapy. Concurrent *TP53* (94%) and *RB1* (94% present either mutation or deletion) inactivation were observed in the NE-like class. Notably, this classifier is limited by the presence of heterogeneous tumors, which may contain multiple subtypes. Further studies are needed to evaluate the impact of intratumor heterogeneity on its accuracy and applicability.

## 5. Molecular Subtyping of NEBC

Koshkin *et al*. analyzed distinct gene expression patterns in SCBC and corresponding normal bladder tissues [45]. Gene expression profiling of 40 tumors plus 6 “normal-appearing” tissues from histologically confirmed 63 SCBC cohort revealed 4 distinct subsets (cluster 1–4) by unsupervised hierarchical clustering. These clusters are associated with discrete clinical phenotypes that correlated with 5-year OS. Cluster 2 (“normal-like”) exhibited the best OS, while Cluster 3 (“metastasis-like”) was associated with a higher likelihood of metastasis and shorter OS (6 months). In multivariate analysis, DLL3 and CD56/NCAM1 expression in tumors were identified as negative prognostic biomarkers for SCBC. The efficacy of DLL3-targeting antibody-drug conjugates (ADCs) was validated in an SCBC PDX model, highlighting DLL3 as a potential therapeutic target in SCBC.

Using the cluster analysis for lineage-specific transcription factors (TFs) identified in SCLC [53,54], 44 SCBC cases were classified into three subtypes (ASCL1, NEUROD1, and POU2F3) and validated their respective downstream targets [38]. Specifically, mixed cases were enriched in the POU2F3 subtype. Heterogeneity in the immune marker expression in these subtypes may suggest their different response to immunotherapy. The NE markers expression-low POU2F3 subtype expressed the highest immune signature scores and the NE markers expression-high ASCL1 subtype significantly expressed CEACAM5, suggesting a potential response to CEACAM5-specific antibody-drug conjugate (ADC) - labetuzumab govitecan, currently under clinical investigation.

Recently, IHC for ASCL1, NEUROD1, and POU2F3 has been integrated into NEBC subtyping. Based on the analysis of the IHC co-expression pattern of ASCL1, NEUROD1, and POU2F3 in 116 SCBC/LCNBC tumors, 5 subtypes were identified as ASCL1+ (34%), NEUROD1+ (23%), ASCL1+/NEUROD1+ (16%), POU2F3+ (21%), ASCL1−/NEUROD1− (6%) [39]. These molecular subtypes have no specific microscopic H&E features to be distinguished reliably by histomorphology. Most POU2F3+ subtype tumors lacked traditional NE markers expressions (SYP, CHGA, INSM1) and showed reduced CD56 expression, in contrast to the ASCL1 and NEUROD1 subtypes. Furthermore, DLL3, a therapeutic target for antibody-drug conjugates or bispecific T-cell engagers, is highly expressed in 82% of SCBC cases. POU2F3+ and PLCG2+ (a top upregulated gene in SCLC associated with pro-metastatic and stem cell-like features [55]) are significantly associated with worse patient outcomes in the radical cystectomy cohort.

## 6. Tumor Heterogeneity in NEBC

Tumor heterogeneity plays an important role in tumor progression, resistance to treatment, and metastasis [56–58]. The complexity of heterogeneity includes clonal expansion of individual mutations, genomic alterations, and expression differences within different patient tumors (interpatient heterogeneity), different regions in one tumor (intratumoral heterogeneity), primary tumor and its metastatic tumor (intertumoral heterogeneity), and over time changing tumor (temporal heterogeneity) [59,60]. MIBC is a highly heterogeneous disease [61]. However, within MIBC, tumor heterogeneity in NEBC has not been systematically investigated.

At the level of histological morphology, within SCBC [62] or LCNEC [24], clinicopathologic differences were observed between tumors, including histopathology and immunohistochemistry, as well as incidence differences of mixed UC patterns and tumor stages differences. A heterogeneous immunophenotypical profile of urinary NEBC has also been observed [63]. Based on the differential IHC co-expression patterns of ASCL1, NEUROD1, and POU2F3 in 116 NEBC tumors, five distinct NEBC subtypes were identified: ASCL1+, NEUROD1+, ASCL1+/NEUROD1+, POU2F3+, ASCL1−/NEUROD−[39]. Figure 2 also demonstrates mixed UC/SCBC histologic characteristics in 1 out of 3 SCBC patients.

At the mutational level, although the three most frequent mutations (*TP53*, *RB1*, and *TERT* promoter) are common, individual tumors from patients exhibited distinct mutation patterns. *RB1* mutations are a common hallmark of SCBC; however, some SCBC patients lose RB protein expression despite the absence of detectable *RB1* mutations [7]. Mutations in epigenetic modifiers, such as *KMT2D*, *CREBBP*, *ARID1A*, and *EP300* were frequently altered [7,34]. However, these chromatin-remodeling genes were not frequently altered in another SCBC cohort [37]. *ERCC2* mutations in some SCBC tumors are associated with improved responses to NAC [34].

At a transcriptomic level, molecular subtyping of SCBC has been studied for patient stratification. The distinct gene expression patterns within SCBC divided tumors into 4 distinct clusters [45], and the “metastasis-like” cluster was associated with shorter OS. A recent study grouped SCBC cases into three subtypes (ASCL1, NEUROD1, and POU2F3) using cluster analysis for lineage-specific TFs [38]. The POU2F3 subtype with low expression of NE markers exhibited the highest immune signature scores. Overall, the heterogeneity in NEBC remains understudied. A deeper understanding of NEBC heterogeneity could aid in developing more precise therapies, ultimately improving patient outcomes.

## 7. Cellular Plasticity of NEBC

Lineage plasticity refers to the ability of cells to reprogram and change to a different phenotypic identity [58,64]. These changes often involve transcriptional or epigenetic fluctuations, and occasionally genetic alterations, which lead to cell state plasticity to promote tumor initiation, progression, metastasis, immune evasion, and chemoresistance [65,66]. Few studies have investigated lineage plasticity in NEBC, but existing research suggest possible UC-to-NEBC plasticity. No conclusive evidence of NEBC-to-UC plasticity has been reported.

A comprehensive genomic profiling analysis of genitourinary NE (11 SCBC and 1 prostate small cell carcinoma resected from the bladder) was conducted [42]. Consistently, frequent alterations of *TP53*, *RB1*, *PIK3CA*, *ERCC2*, *ARID1A*, and *EP300* were detected in SCBC, which were detected in UC as well [67,68]. Integrated analysis revealed three key altered function modules, including the p53/Rb pathway, receptor tyrosine kinase signaling, and epigenetic regulators. Together with the related works [7,69,70], NE and UC were proved to share generally identical genotypic and etiological characteristics, which supported that they share a common cell origin. Furthermore, preliminary results from CRISPR experiments targeting *TP53* and *RB1* in UC cell lines TCCSUP and J82 revealed an increase in multiple canonical NE markers, suggesting that the loss of TP53 and RB1 may drive a UC-to-NEBC lineage plasticity. This NE lineage plasticity has been also observed in EGFR mutant lung adenocarcinoma [71] and neuroendocrine prostate cancer (NEPC) [72,73].

Another study investigating microRNA-145 in human UC cell lines T24 and KU7 provided evidence supporting cell plasticity within UC tumors [45]. The overexpression of microRNA-145 increased stem cell markers and upregulated markers of squamous, glandular, and NE cells, suggesting that microRNA-145 could reprogram UC cells into multipotent stem-like cells, which subsequently differentiate into other lineages, including squamous, glandular, or NE. However, these studies are limited *to in vitro* molecular analyses and lack *in vivo* validation through NE marker staining and/or histological confirmation of NEBC morphology.

Wang *et al*. reported that EPCAM+/CD49f+ UC cells from the primary human renal pelvis or bladder were transformed into SCBC features by transducing a dominant-negative form of TP53, myr-AKT1, RB1 shRNA, C-MYC, and BCL2, collectively termed PARCB [74]. This artificial SCBC tumor displays diverse histological features, including SCBC, UC, and squamous differentiation. Single clones from bladder SCBC tumors exhibited both SCBC and UC phenotypes, highlighting their plasticity. Transcriptional profiling of 9 SCBC and 10 non-SCBC patient tumor samples revealed that SCBC possesses a distinct and unique transcriptional regulatory network. Cell surface proteins associated with the SCBC phenotype showed similarities between PARCB tumors and clinical SCBC tumors. However, the PARCB construct was initially designed to transform prostate/lung cells into prostate/lung NE tumors. This PARCB model does not necessarily recapitulate human NEBC biology, as PARCB is not naturally present in human NEBC tumors. Nevertheless, the results of PARCB transformation support the possibility of the UC-to-NEBC hypothesis.

In a cohort of 34 SCBCs and 84 conventional UCs along with a reference of 408 MIBCs in the TCGA database [37], four groups (Luminal UC, Basal UC, Double-negative UC, and SCBC) were categorized well by unsupervised hierarchical clustering and whole-transcriptome profiles. The SCBC subtype demonstrated significant enrichment of genes associated with promoting cancer metastasis and activating canonical pathways that suppress adaptive immunity. The expression profile change resulted in the lineage plasticity of SCBC, characterized by global downregulation of urothelial differentiation genes and upregulation of NE differentiation programs. The transcriptome analysis data indicated that UC-to-SCBC lineage plasticity was driven by a dysregulated epithelial-to-mesenchymal (EMT) transition combined with a urothelial-to-neural phenotypic switch.

## 8. Cell of Origin of NEBC

The origin of NEBC has not been definitively determined. Currently, the following hypotheses of cell origin were proposed: 1. pre-existing NE/enterochromaffin cells in the submucosa or normal urothelium; 2. multipotent urothelial stem cell; 3. urinary tract epithelial metaplasia; 4. transformation from UC [30,75]. SCBC closely resembles SCLC in mutation profiles, histological features, and transcriptional patterns. Most lineage tracing studies in SCLC suggest that normal pulmonary NE cells are the cells of origin for SCLC [76]. Therefore, an NE cell origin is generally favored because of the presence of NE cells in the normal urothelium [6]. However, no direct evidence has been reported to confirm that NEBC originates from normal NE cells in the urothelium. The hypothesis of a multipotent stem cell origin is supported by a study showing that microRNA-145 reversed UC in multipotent stem cells, enabling their subsequent differentiation into squamous, NE, and glandular lineages [45]. However, several lines of evidence suggest that NEBC originates from the UC precursor. Most SCBC cases coexist with conventional UC, suggesting that SCBC and UC may share a common clonal origin [63]. This aligns with genetic evidence showing that SCBC and coexisting UC exhibit nearly identical allelic loss patterns [69]. *In vitro* transduction of five genetic factors (PARCB) successfully transformed human urothelial cells into NEBC tumors, suggesting urothelial cells can be transformed into NEBC cells [74]. Nevertheless, the cell of origin for NEBC remains unclear. Further genetic and molecular studies, along with the development of NEBC preclinical models, are needed to clarify its tumor origin, as this will have significant implications for clinical treatment and management.

## 9. Potential Therapeutic Targets Identified by Preclinical NEBC Models

Preclinical models are essential for studying tumor progression and evaluating *in vitro* and *in vivo* efficacy and safety of potential therapeutics prior clinical trials [77–80]. A NEBC GEMM (*Trp53*^*fl/fl*^ *Rb1*^*fl/fl*^ *Myc*^*LSL/LSL*^) was generated using Lenti-sgNeo#2/Cre delivered via transurethral catheterization, resulting in tumors with SCBC histology [81]. In addition, our lab recently developed another mouse NEBC model (*Trp53*^*fl/fl*^ *Rb1*^*fl/fl*^ *Pten*^*fl/fl*^) using Adenovirus-Krt5-Cre. This model produced tumors with NE histology, which were subsequently used to establish stable organoids for drug screening purposes [82]. However, no spontaneous NEBC genetically engineered mouse models (GEMMs) are currently available to study tumor origin, progression, heterogeneity, drug responses, or mechanisms of resistance in NEBC.

Serum-free NEBC cell lines and PDX models were established from two NEBC patients who had not received neoadjuvant treatment [83]. The *in vitro* growth of NEBC spheroids was found to depend on hepatocyte growth factor (HGF). Treatment with PHA-665752, a MET (*MET* encoding HGF receptor) inhibitor, significantly reduced NEBC tumor growth. Another PDX (BL100) was developed from a high-grade NEBC on NSG mice [45]. The BL100 PDX model was utilized to evaluate the preclinical efficacy of Rovalpituzumab tesirine (Rova-T), a novel antibody-drug conjugate designed to target DLL3 expression on the surface of SCBC tumor cells by delivering a cytotoxic agent. Rova-T effectively targets DLL3-expressing tumor initiating cells in the BL100 PDX model. Additionally, two of the three SCBC PDXs established in our lab (Figure 2) successfully recapitulated the histology and molecular profiles of SCBC. These PDXs are currently being utilized for *in vivo* and *in vitro* drug treatment studies and to evaluate novel therapeutic combinations. These relevant cell lines, GEMMs, and PDX models will serve as valuable tools for studying the tumor origin, progression, heterogeneity, drug response, and resistance in NEBC.

## 10. Conclusions and Perspectives

NEBCs are rare, accounting for approximately 1% of bladder cancers, with most cases represented as SCBC. SCBC shared some pathogenic features with SCLC but remains distinct in mutational burden driven predominantly by APOBEC-mediated mutational process and diverse epigenetic modifiers [7]. *TP53* and *RB1* co-mutations are the most common alterations in SCBC. RB1 protein loss is observed in some tumors with the wild-type *RB1* gene. Bladder-specific mutations in the *TERT* promoter and chromatin-modifying genes (e.g., *KMT2D*, *CREBBP*, *ARID1A*, *EP300*) are also identified in NEBC. *ERCC2* mutations, a DNA repair gene, are associated with a complete response to NAC.

NEBC displays significant tumor heterogeneity at the transcriptional level and can be categorized by different classifiers. A unified consensus subtyping system of 6 classifiers for MIBC was recently developed [44], however, many NE-like tumors didn’t exhibit the NE histologically. A single-sample transcriptomic classifier provides a more stringent and accurate definition of the NE-like subtype [43]. Within histologically confirmed NEBC, 4 distinct subsets have been developed by unsupervised hierarchical clustering [45]. Cluster analysis for lineage-specific TFs divided SCBC into three subtypes (ASCL1, NEUROD1, and POU2F3) [38]. This lineage-specific subtyping system was expanded by incorporating the IHC expression patterns of ASCL1, NEUROD1, and POU2F3, resulting in the identification of five distinct subsets [39].

The tumor heterogeneity and plasticity between NEBC and UC haven’t been systematically investigated. The cell of origin of NEBC remains unknown. Recent evidence suggests that SCBC may share a common origin with UC, arising from a UC precursor through UC-to-NEBC plasticity. However, there is also a possibility that NEBC originates from normal NE cells or stem cells within the urothelium. GEMMs of SCLC suggest that both the cell of origin and the genetic driver mutations play a critical role in shaping the phenotypes of SCLC tumors [84]. Hence, GEMMs of SCBC hold promise for providing insights into the cells of origin and identifying the driver lesions and/or epigenetic modifications required for cellular plasticity into NEBC. Additional PDX models will enhance our understanding of NEBC biology and support the development of more effective therapies.

Despite recent advances and collaborative efforts, NEBC continues to present significant challenges and opportunities: 1. identify and validate targetable driver mutations for discovering new therapeutics targets for NEBC; 2. develop more reliable and stable preclinical models (cell line or mouse models) with NEBC features. 3. determine the cell of origin of NEBC with more *in vitro* and in *vivo* evidence; 4. understand the mechanism of cell plasticity between UC and NEBC; 5. systemic study and analysis on heterogeneity in NEBC; 6. precisely classify patient samples with NE or NE-like features to select the optimal treatment. Future advances in basic and translational research present promising opportunities to improve outcomes for NEBC patients.

## Figures and Tables

**Table 1.** Summary of molecular characterization studies on NEBC.

Cohort	Source	Genomic Seq	RNA Seq	Genomic Mutation	Key Findings	Citations
307 advanced UC	Lund University	N/A	47 Sc/NE	N/A	Lund subtyping taxonomy; mRNA profiling-6 clusters; IHC profiling-5 subtypes; **Sc/NE**	2017 Sjodahl *et al*. [40]
412 MIBC	TCGA	20 neuronal	20 neuronal	*TP53* 65%, *RB1* 25%	Five subtypes: luminal-papillary, luminal-infiltrated, luminal, basal-squamous, and **neuronal**	2017 Robertson *et al*. [41]
12 SCBC	Shanghai Jiao Tong University	12 SCBC	12 SCBC	*TP53* 83%, *RB1* 33%	CRISPR in J82 and TCCSUP increased canonical NE markers	2018 Shen *et al*. [42]
61 SCBC	MSKCC	61 SCBC	12 SCBC	TP53 90%, RB1 90%, co-occurred 80%, TERT promoter 95%	SCBC convergent distinct pathogenesis with SCLC	2018 Chang *et al*. [7]
PTC, RC1, RC2, NAC, TMT, Imvigor210	University of British Columbia	N/A	34 NE-like	N/A	A strict and accurate single-patient classifier for **NE-like**	2019 Batista *et al*. [43]
1750 MIBC	Multi-centers	44 NE-like	44 NE-like	NE-like: *TP53* 94%, *RB1* 39% (94% present either mutation or deletion)	Consensus six subtypes: luminal papillary, luminal non-specified, luminal unstable, stroma-rich, basal/squamous, and **NE-like**	2019 Kamoun *et al*. [44]
63 SCBC	Cleveland Clinic	N/A	40 SCBC 6 normal	N/A	First subtyping for SCBC, including 4 distinct subsets; DLL3 as a prognostic marker	2019 Koshkin *et al*. [45]
34 SCBC 84 UC	MD Anderson	34 SCBC 84 UC	34 SCBC 84 UC	*TP53* 93%, *RB1* 47%,	Identical TP53 mutation in paired UC and SCBC; UC-to-NE plasticity drive SCBC	2020 Yang *et al*. [37]
132 SCBC 3236 UC	Foundation Medicine, Inc.	132 SCBC	24 SCBC 51 UC	*TP53* 92%, *RB1* 75%, co-occurred 72%, *TERT* promoter 68%	Low T-cell biomarkers; Suppression of inflammatory pathway	2021 Hoffman *et al*. [32]
43 LCNEC 192 SCBC	MSKCC	18 LCNEC 52 SCBC	N/A	LCNEC: *TP53* 100%, *RB1* 100%, co-occurred 56%, *TERT* promoter 89%	LNEC longer OS compared with SCBC-only	2021 Guercio *et al*. [25]
199 SCBC	MSKCC	47 SCBC	N/A	*TP53* 87%, *RB1* 79%, *TERT* promoter 83%	*ERCC2* mutation associated with NAC response	2022 Teo *et al*. [34]
80 LCNEC	MSKCC	37 LCNEC	N/A	*TP53* 92%, *RB1* 65%, *TERT* promoter 76%	Similar genomic profiles of LCNEC and SCBC	2023 Gandhi *et al*. [26]
44 SCBC	Johns Hopkins Medical Institutions	24 SCBC	44 SCBC	*TP53* 75%, *RB1* 38%, *TERT* promoter 92%	Cluster analysis for lineage-specific TFs (ASCL1, NEUROD1, and POU2F3); high ASCL1 subtype expressed CEACAM5	2024 Feng *et al*. [38]
103 SCBC 19 LCNBC	MSKCC	33 SCBC/LCNBC	N/A	*TP53* 91 %, *RB1* 79%, *TERT* promoter 82%	5 subtypes analysis by IHC co-expression pattern of ASCL1, NEUROD1, and POU2F3; DLL3 a therapeutic target	2024 Akbulut *et al*. [39]

## Abbreviations

NEBC: neuroendocrine bladder cancer
SCBC: small cell bladder cancer
UC: urothelial carcinoma
SCLC: small cell lung cancer
MIBC: muscle-invasive bladder cancer
WHO: World Health Organization
NE: neuroendocrine
LCNEC: large cell neuroendocrine carcinoma
TURBT: transurethral resection of the bladder tumor
NSE: neuron-specific enolase
INSM1: Insulinoma-associated protein 1
EMA: epithelial membrane antigen
NAC: neoadjuvant chemotherapy
TCGA: The Cancer Genome Atlas
UNC: University of North Carolina
MDA: MD Anderson Cancer Center
H&E: hematoxylin and eosin
IHC: immunohistochemistry
SYP: synaptophysin
CHGA: chromogranin-A
ADC: antibody-drug conjugate
PDX: patient-derived xenograft
TFs: transcription factors
CRISPR: clustered regularly interspaced short palindromic repeats
PARCB: a dominant-negative form of TP53 (TP53-DN), myristoylated AKT1 (myr-AKT1), RB1 short-hairpin RNA, C-MYC, and BCL2
MSKCC: Memorial Sloan Kettering Cancer Center
OS: overall survival
SCCL: squamous cell carcinoma-like
HGF: hepatocyte growth factor
Rova-T: Rovalpituzumab tesirine
GEMMs: genetically engineered mouse models
RPCCC: Roswell Park Comprehensive Cancer Center
